# Risk Factors for Migration, Fracture, and Dislocation of Pancreatic Stents

**DOI:** 10.1155/2015/365457

**Published:** 2015-04-05

**Authors:** Yoshiaki Kawaguchi, Jung-Chun Lin, Yohei Kawashima, Atsuko Maruno, Hiroyuki Ito, Masami Ogawa, Tetsuya Mine

**Affiliations:** Department of Gastroenterology, Tokai University School of Medicine, Isehara 259-1193, Japan

## Abstract

*Aim*. To analyze the risk factors for pancreatic stent migration, dislocation, and fracture in chronic pancreatitis patients with pancreatic strictures. *Materials and Methods*. Endoscopic stent placements (total 386 times) were performed in 99 chronic pancreatitis patients with pancreatic duct stenosis at our institution between April 2006 and June 2014. We retrospectively examined the frequency of stent migration, dislocation, and fracture and analyzed the patient factors and stent factors. We also investigated the retrieval methods for migrated and fractured stents and their success rates. *Results*. The frequencies of stent migration, dislocation, and fracture were 1.5% (5/396), 0.8% (3/396), and 1.2% (4/396), respectively. No significant differences in the rates of migration, dislocation, or fracture were noted on the patient factors (etiology, cases undergoing endoscopic pancreatic sphincterotomy, location of pancreatic duct stenosis, existence of pancreatic stone, and approach from the main or minor papilla) and stent factors (duration of stent placement, numbers of stent placements, stent shape, diameter, and length). Stent retrieval was successful in all cases of migration. In cases of fractured stents, retrieval was successful in 2 of 4 cases. *Conclusion*. Stent migration, fracture, and dislocation are relatively rare, but possible complications. A good understanding of retrieval techniques is necessary.

## 1. Introduction

Endoscopic pancreatic stenting using a plastic stent is currently a well-accepted treatment for pancreatic duct stenosis, secondary to chronic pancreatitis [1–4]. The reported complication rate associated with the use of pancreatic stents ranges from 4% to 10% [5–9], and the complications described include pancreatitis, duodenal perforation, bleeding, stent fracture, proximal migration, distal dislocation, and occlusion, resulting in recurrent pancreatic obstruction [1–9]. Stent migration, fracture, and dislocation are relatively rare complications associated with pancreatic stenting [3, 4]. Most plastic stents are straight or sigmoid shaped with flaps at each end, to prevent migration or dislocation. However, stents may migrate proximally or dislocate distally as a late complication of endoscopic stenting in approximately 4–10% of patients who have undergone pancreatic duct stenting [5–9]. Stent fracture can occur at the time of stent removal, as a result of severe pancreatic duct stenosis in chronic pancreatitis patients. These late complications can cause pancreatic duct obstruction and pancreatitis, thus requiring stent retrieval or restenting. Therefore, it is important to determine the factors influencing pancreatic plastic stent migration, dislocation, and fracture. Several studies have previously reported the risks associated with these complications [1–10].

The retrieval of migrated or fractured stents is technically challenging, but can usually be achieved endoscopically, using forceps, snare, or balloon techniques, and rarely requires surgical intervention [7, 10–13]. In the present study, we aimed to determine the frequency of pancreatic duct stent migration, dislocation, and fracture; analyze the risk factors for these complications; and describe the methods used for retrieval of migrated or fractured stents.

## 2. Patients and Methods

### 2.1. Patients

Between April 2006 and June 2014, endoscopic stent placement was performed in 99 chronic pancreatitis patients with pancreatic duct stenosis at our institution (the total number of stents inserted for them in this period was 386). The mean patient age was 58.3 years, and 78% of the patients were male. Disease etiology included alcoholism in 89, pancreatic divisum in 2, post-pancreatectomy in 1, and idiopathic disease in 7 patients (Table 1).

### 2.2. Endoscopic Therapy

Endoscopic retrograde cholangiopancreatography (ERCP) was performed using a JF-240, JF-260V, or TJF-260V unit (Olympus Medical Systems Corp., Tokyo, Japan) with the patient under conscious sedation using diazepam and pethidine. After a guide wire (Jagwire High Performance Guidewire, Boston Scientific Corp., Natick MA, USA or VisiGlide, Olympus Medical Systems Corp.) was successfully placed across the stenotic lesion, brushing cytology, pancreatic juice cytology, or biopsy was performed to diagnose the disease on a case-by-case basis.

### 2.3. Dilation of Pancreatic Duct Stenosis

In the case of severe stenosis, the stenotic lesions were dilated with a dilation catheter (6-, 7-, or 9-Fr, Soehendra Biliary Dilation Catheter, Cook Medical, Bloomington, IN, USA) or a dilation balloon catheter (diameter: 6 mm, length: 2 cm; Hurricane RX Balloon Dilation Catheter, Boston Scientific Corp.).

### 2.4. Pancreatic Duct Stenting

After dilation, a straight stent (Cook Medical) or sigmoid stent (Olympus Medical Systems Corp.) was implanted in the stenotic lesion. These were 5- or 10-Fr polyethylene stents with multiple side holes.

A pancreatic duct stent was implanted to drain the pancreatic juice and dilate the stenotic lesions of the pancreatic duct.

### 2.5. Stent Migration

We have defined stent migration in the present study as proximal migration of the stent into the pancreatic duct.

### 2.6. Stent Dislocation

We have defined stent dislocation in the present study as distal migration of the stent into the duodenum.

### 2.7. Stent Fracture

We have defined stent fracture in the present study as a split in the stent identified at the time of stent removal.

### 2.8. Retrieval of Migrated or Fractured Stents

We retrieved the migrated or fractured stents using a basket catheter (4 wires or 8 wires), snare catheter, rat-toothed forceps, biopsy forceps, balloon catheter, or stent retriever.

### 2.9. Patient, Stent, and Retrieval Factors Analyzed

We retrospectively examined the frequencies of stent migration, dislocation, and fracture and analyzed patient characteristics (etiology, whether endoscopic pancreatic sphincterotomy (EPST) was performed, location of pancreatic duct stenosis, existence of pancreatic stone, and approach from main or minor papilla) and stent factors (duration of stent placement, number of stent placements, and stent type, diameter, and length). We also investigated the retrieval methods for migrated stents and their rates of success.

### 2.10. Statistical Analysis

Results were expressed as means (standard deviations, SD) or as a percentage of the total number of patients. The Mann-Whitney *U* test and the chi-square test were used to compare differences between 2 groups. Statistically, a *P* value of <0.05 was considered significant. All analyses were performed using Stat View statistical software Version 5.0 (SAS Institute, Cary, NC, USA).

## 3. Results

### 3.1. Frequencies of Stent Migration, Dislocation, and Fracture

The frequencies of stent migration, dislocation, and fracture were 1.5% (5/396), 0.8% (3/396), and 1.2% (4/396), respectively (Table 1).

### 3.2. Patient Factors

#### 3.2.1. Etiology

Alcoholism was the etiology in 4 of 5 (80%), 3 of 3 (100%), and 3 of 4 (75%) cases associated with stent migration, dislocation, and fracture, respectively (Tables 3, 6, and 8). The overall number of cases associated with alcoholism in this series was 89/99 (90%) (Table 1). The frequencies of migration, dislocation, and fracture were not significantly higher in cases with an etiology of alcoholism than in those with other etiologies.

#### 3.2.2. Cases Undergoing Endoscopic Pancreatic Sphincterotomy

No patients with stent migration, dislocation, or fracture had previously undergone EPST (Tables 3, 6, and 8). The overall number of patients who had undergone EPST in this series was 9/99 (9.1%) (Table 1). The frequencies of migration, dislocation, and fracture were not significantly higher in patients who had undergone EPST than in patients who had not undergone EPST.

#### 3.2.3. Location of Pancreatic Duct Stenosis

Stenosis of the pancreatic head was noted in all cases of stent migration, dislocation, and fracture (Tables 3, 6, and 8). Of the total number of patients in the series, stenosis of the pancreatic head was noted in 88 of 99 (88.9%) cases (Table 1). The rates of migration, dislocation, and fracture were not significantly higher in cases with stenosis of the pancreatic head than in cases with other stenotic lesions.

#### 3.2.4. Existence of Pancreatic Stones

The existence of pancreatic stones was noted in 2 of 5 (40%), 2 of 3 (66.7%), and 3 of 4 (75%) cases with stent migration, dislocation, and fracture, respectively (Tables 3, 6, and 8). The total number of cases with pancreatic stones in this series was 75/99 (75.8%) (Table 1). The frequencies of migration, dislocation, and fracture were not significantly higher in cases with pancreatic stones than in cases without pancreatic stones.

#### 3.2.5. Approach from the Main or Minor Papilla

An approach from the main papilla was carried out in 4 of 5 (80%), 3 of 3 (100%), and 3 of 4 (75%) cases with stent migration, dislocation, and fracture, respectively (Tables 3, 6, and 8). The total number of stentings with an approach from the main papilla in this series was 274/386 (71%) (Table 2). The rates of migration, dislocation, and fracture were not significantly higher in cases using an approach from the main papilla compared to cases using an approach from the minor papilla.

### 3.3. Stent Factors

#### 3.3.1. Duration of Stent Placement

Stent placement duration of >3 month was noted in 4 of 5 (80%), 1 of 3 (33.3%), and 3 of 4 (75%) cases with stent migration, dislocation, and fracture, respectively (Tables 4, 7, and 9). In 1 case, the stent migrated at the time of stent placement. The number of patients with stent placement duration of >3 month in the series was 363/386 (94%) (Table 2). The frequency of migration was not significantly higher in cases with a stent placement duration of >3 months than in cases with a stent placement duration of ≤3 months.

#### 3.3.2. Numbers of Stent Placements

Number of stent placements ≤4 was noted in 3 of 5 (60%), 2 of 3 (66.7%), and 3 of 4 (75%) cases with stent migration, dislocation, and fracture, respectively (Tables 4, 7, and 9). The number of patients with ≤4 stent placements in the series was 258/386 (66.8%) (Table 2). The rate of migration was not significantly higher in cases with ≤4 stent placements than in cases with >4 stent placements.

#### 3.3.3. Stent Shape

Straight-type stents were used in 5 of 5 (100%), 1 of 3 (33.3%), and 4 of 4 (100%) cases associated with migration, dislocation, and fracture, respectively (Tables 4, 7, and 9). The total number of patients who received straight-type stents was 312/386 (80.8%) (Table 2). The rate of migration was not significantly higher in cases using straight-type stents than in cases using S-shaped type stents.

#### 3.3.4. Stent Diameter

Stent migration occurred in 1 of 5 (20%) and 4 of 5 (80%) patients who had received a 10-Fr-sized stent and 7-Fr-sized stent, respectively (Table 4). Stent dislocation was reported in 2 of 3 (66.7%) and 1 of 3 (33.3%) patients who had received an 8.5-Fr-sized stent and 7-Fr-sized stent, respectively (Table 7). Stent fracture occurred in 3 of 4 (75%) and 1 of 4 (25%) patients who had received a 7-Fr-sized stent and 5-Fr-sized stent, respectively (Table 9). The total numbers of patients who received 10-Fr, 8.5-Fr, 7-Fr, and 5-Fr-sized stents were 36/386 (9.3%), 84/386 (21.8%), 168/386 (43.5%), and 98/386 (25.4%), respectively. When classifying 5-Fr and 7-Fr stents as thin stents and 8.5-Fr and 10-Fr stents as thick stents, the total numbers of patients who received thin stents and thick stents were 266/386 (68.9%) and 120/386 (31.1%), respectively (Table 2). The frequency of migration, dislocation, or fracture was not significantly higher using thick stents than using thin stents.

#### 3.3.5. Stent Length

As stents of various lengths were used, we classified stents ≤7 cm as short stents and those ≥8 cm as long stents. Short and long stents were used in 2 of 5 (40%) and 3 of 5 (60%) cases of stent migration, respectively (Table 4). Short and long stents were used in 1 of 3 (33.3%) and 2 of 3 (66.7%) cases of stent dislocation, respectively (Table 7), and short and long stents were used in 3 of 4 (75%) and 1 of 4 (25%) cases of stent fracture, respectively (Table 9). The total numbers of patients who received short and long stents were 266/386 (69.0%) and 120/386 (31.0%), respectively (Table 2). No significant differences in the rates of migration, dislocation, or fracture were noted between patients who received short and long stents.

### 3.4. Retrieval Methods for Migrated and Fractured Stents and Their Success Rates

**Figure 1:**
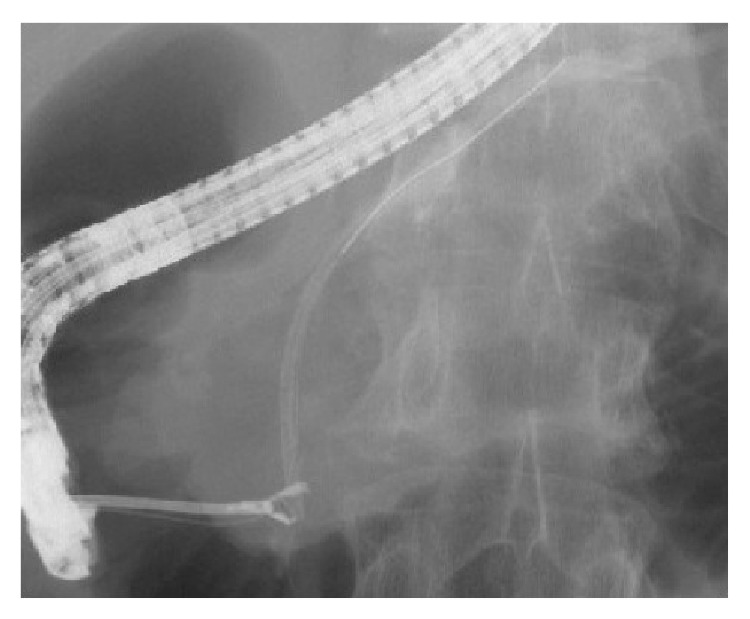
This retrieval technique is a method that directly grasps the distal end of the migrated stent using a rat-toothed forceps.

**Figure 2:**
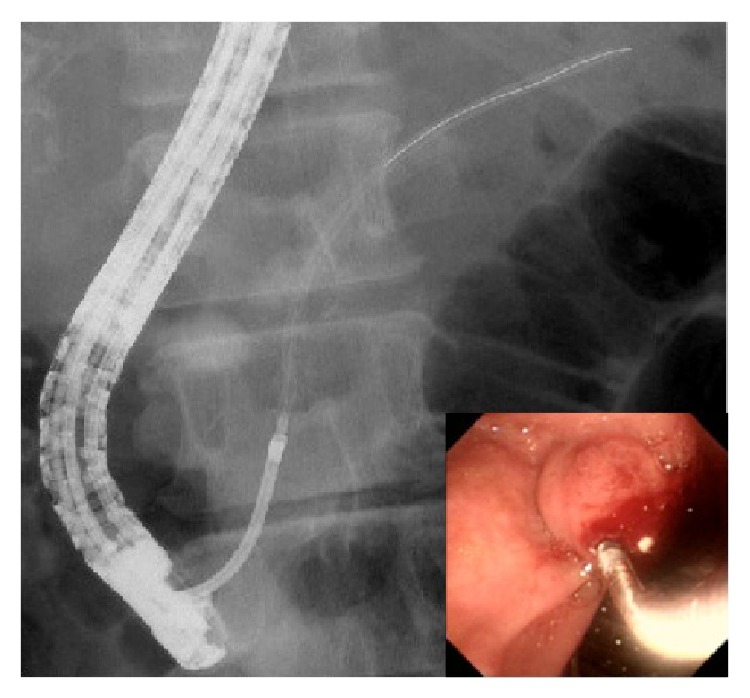
This retrieval technique is a method carried out by connecting the distal end of the migrated stent to a stent retriever using a guide wire passed through the lumen of the migrated stent.

The grasping technique was used in 2 of 5 (40%) cases of stent migration (Table 5). This is a retrieval technique that directly grasps the distal end of the stent using a basket, snare, rat-toothed forceps, or biopsy forceps (Figure 1). The cannulation technique was used in 3 of 5 (60%) cases of stent migration (Table 10). This retrieval technique is carried out by connecting the distal end of the stent to a stent retriever, balloon catheter, or a cannula using a guide wire passed through the lumen of the migrated stent (Figure 2). Stent retrieval was successful in all cases of migration (Table 5). In cases of fractured stents, retrieval was successful in 2 of 4 cases (Table 10). The grasping technique, using a snare, was used in both of these cases. The stent was not retrieved in the other 2 cases. In 1 case we inserted another stent, and in the other case we continued follow-up without the insertion of further stents.

## 4. Discussion

Endoscopic stent placement for the treatment of main pancreatic duct stenosis is less invasive than surgical pancreatoenteric anastomosis, and its utility has occasionally been reported [1, 2]. However, periodic stent exchange is necessary because of stent deterioration or obstruction, and complications associated with stent placement have been reported. The reported complications of pancreatic stenting include pain associated with stent placement, stent obstruction, obstruction of a pancreatic duct branch, pancreatitis, or pancreatic abscess associated with obstruction of the stent, pancreatic duct mucosal injury, dislocation, caudal migration, difficult removal, stent fracture during removal, stent-related duodenal erosion and ulcer, duodenal perforation, and bleeding [3, 4]. Of these, pancreatic stent migration is regarded as an important complication with a relatively rare frequency (4–10%) [5–9]. In this study, the frequency of migration was found to be 1.5%, slightly lower than figures reported in previous studies. The frequencies of dislocation and fracture during removal were found to be 0.8% and 1.2%, respectively.

The major causes of these complications include stent destruction and inappropriate placement [5, 10]. Another possible explanation for the occurrence of complications is that stent placement to suit individual pancreatic ducts is difficult due to limitations in the shapes of commercially available stents. In this study, the following patient factors were examined: the etiology of pancreatitis, site of stenosis, existence of pancreatic stones, incision in the opening of the pancreatic duct, and approach from the main or minor papilla. In addition, stent diameter, length, and shape and duration and number of stent placements were examined as stent factors. Although risk factors for migration were not identified in this study, due caution is necessary in patients with prolonged stent placement, or those in whom the stent has frequently been exchanged. Risk factors for dislocation were not identified, but the possibility of dislocation should be considered when stenting is performed in patients with prolonged stent placement or those in whom stenosis has been eliminated. Although risk factors for fracture during removal also remain to be identified, stent fracture is possible at the time of removal in cases with calcified pancreatic stones in the main pancreatic duct (following extracorporeal shock wave lithotripsy) or when a thin stent (5–7 Fr) is used.

Retrieval of migrated stents is often difficult. As migration of a pancreatic duct stent may cause pancreatitis, pancreatic abscess, or pancreatic duct stenosis or perforation, it is necessary to retrieve the migrated stent as soon as possible [10, 14, 15]. Endoscopic techniques should be attempted initially. If these techniques are unsuccessful, surgical techniques may be needed. In this study, the rate of successful retrieval of migrated stents was 100%. On the contrary, fragments of stents fractured during removal were difficult to retrieve due to the presence of severe stenosis (success rate, 50%).

Endoscopic retrieval of migrated pancreatic stents uses instruments, such as biopsy forceps [7, 10–13], basket catheters [7, 11, 12], snare catheters [7, 10–12], balloon catheters [7, 11, 12], and stent retrievers [7]. The method of retrieval is basically the same as that for the retrieval of biliary stents. However, it should be noted that manipulation of these instruments is difficult as the pancreatic duct is curved and meandering and thinner than the biliary tract. Briefly, the technique involves identification of the pancreatic duct with a guide wire and straightening of the main pancreatic duct by passing the guide wire through the stenotic part. The subsequent procedure, such as targeting the stent lumen using a second guide wire, should follow. When a stent migrates caudally, in cases of severe stenosis, it is particularly difficult to insert a retrieval instrument and pass the retrieved stent through the stenotic part. When retrieval is difficult, a second stent should be placed to avoid subsequent complications, such as pancreatitis. Fractured stents are difficult to retrieve as the fractures occur at the site of stenosis. In both of our cases of successful retrieval, a guide wire was first passed through the stenotic part, and the stent was retrieved with a guide-wire-led snare. A stent exchange was carried out in 1 of the 2 unsuccessful cases. The other case was associated with pancreatic stone congestion and is currently being followed up without stent retrieval.

## 5. Conclusion

Stent migration, fracture, and dislocation are possible complications. Though we could not identify these risk factors, we recommend exchanging the stent for short time to avoid them. A good understanding of retrieval techniques is necessary, particularly for cases of difficult retrieval.

## Figures and Tables

**Table 1:** Demographic characteristics of the study population.

	*n* = 99
Gender	
Male	77 (78%)
Female	12 (12%)
Age; years (mean ± SD)	58.3 ± 14.5
Etiology	
Alcohol	89 (80%)
Pancreatic divisum	2 (2%)
Post-pancreatectomy	1 (1%)
Idiopathic disease	7 (7%)
EPST	9 (9.1%)
Location of pancreatic duct stenosis	88 (88.9%)
Existence of pancreatic stone	75 (75.8%)

**Table 2:** Demographic characteristics of the stent placement.

	*n* = 386
Approach from the main papilla	274 (71%)
Stent placement duration of >3 month	363 (94%)
Number of patients with ≤4 stent placements	258 (66.8%)
Stent shape	
Straight-type stent	312 (80.8%)
S-shaped stent	64 (19.2%)
Stent diameter	
Thin stent (5, 7 Fr)	266 (68.9%)
Thick stent (8.5, 10 Fr)	120 (31.1%)
Stent length	
Short stent (≤7 cm)	266 (69.0%)
Long stent (≥8 cm)	120 (31.0%)

**Table 3:** Patients characteristics of migrated cases.

Case	Age	Sex	Etiology	Location of stenosis	Pancreatic stone	EPST	Approach
1	51	M	Alc.	Head	−	−	Major
2	55	M	Alc.	Head	+	−	Major
3	59	M	Alc.	Head	+	−	Major
4	63	M	Alc.	Head	−	−	Major
5	67	F	Divisum	Head	−	−	Minor

**Table 4:** Stents characteristics of migrated cases.

Case	Diameter (Fr)	Length (cm)	Type	Duration of stent placement (Day)	Times of stent placement
1	10	6	Straight	81 (In the time of placement)	3
2	7	7	Straight	115	3
3	7	9	Straight	91	9
4	7	9	Straight	154	7
5	7	5	Straight	88	2

**Table 5:** Stents retrieval of migrated cases.

Case	Retrieval	Method
1	Success	Balloon catheter
2	Success	Rat-toothed forceps
3	Success	Stent retriever
4	Success	Stent retriever
5	Success	Stent retriever

**Table 6:** Patients characteristics of dislocated cases.

Case	Age	Sex	Etiology	Location of stenosis	Pancreatic stone	EPST	Approach
1	50	F	Alc.	Head	+	−	Major
2	51	M	Alc.	Head	+	−	Major
3	51	M	Alc.	Head	−	−	Major

**Table 7:** Stents characteristics of dislocated cases.

Case	Diameter (Fr)	Length (cm)	Type	Duration of stent placement (Day)	Times of stent placement
1	8.5	8	S-shaped	118	1
2	8.5	8	S-shaped	90	2
3	7	7	Straight	64	9

**Table 8:** Patients characteristics of fractured cases.

Case	Age	Sex	Etiology	Location of stenosis	Pancreatic stone	EPST	Approach
1	52	M	Alc.	Head	−	−	Major
2	42	M	Alc.	Head	+	−	Major
3	74	M	Alc.	Head	+	−	Major
4	67	F	Divisum	Head	+	−	Minor

**Table 9:** Stents characteristics of fractured cases.

Case	Diameter (Fr)	Length (cm)	Type	Duration of stent placement (Day)	Times of stent placement
1	7	12	Straight	19	11
2	7	5	Straight	104	3
3	7	5	Straight	102	2
4	5	9	Straight	100	1

**Table 10:** Stents retrieval of fractured cases.

Case	Retrieval	Method	Follow-up
1	Success	Snare	
2	Failure		Without stent
3	Success	Snare	
4	Failure		Second stent
